# Effectiveness of a school-based intervention on knowledge, attitude and practice on healthy lifestyle and body composition in Malaysian adolescents

**DOI:** 10.1186/s12887-020-02023-x

**Published:** 2020-03-14

**Authors:** Sharifah Intan Zainun Sharif Ishak, Yit Siew Chin, Mohd. Nasir Mohd. Taib, Yoke Mun Chan, Zalilah Mohd. Shariff

**Affiliations:** 1 Department of Healthcare Professional, Faculty of Health and Life Sciences, Management and Science University, 40100 Shah Alam, Selangor Malaysia; 2 Department of Nutrition and Dietetics, Faculty of Medicine and Health Sciences, Universiti Putra Malaysia, 43400 Serdang, Selangor Malaysia; 3 Research Centre of Excellence for Nutrition and Non-Communicable Diseases, Faculty of Medicine and Health Sciences, Universiti Putra Malaysia, 43400 Serdang, Selangor Malaysia

**Keywords:** Overweight, Obesity, Adolescent, Intervention, Peer-led, Malaysia, Knowledge, Body composition

## Abstract

**Background:**

The ‘Eat Right, Be Positive About Your Body and Live Actively’ (EPaL) intervention programme was developed to prevent overweight and disordered eating in Malaysian adolescents. This study aimed to evaluate the effectiveness of the EPaL programme on knowledge, attitudes and practices on healthy lifestyle and body composition (body mass index z-score [zBMI], waist circumference [WC] and body fat percentage [BF%]) among adolescents.

**Methods:**

All measures were taken at three time points: before intervention (Pre), after intervention (Post I) and 3 months after intervention (Post II). The intervention group (IG) participated in the EPaL programme for 16 weeks, whereas the comparison group (CG) received no intervention. Seventy-six adolescents (IG: *n* = 34; CG: *n* = 42) aged 13–14 years were included in the final analysis. Repeated measures analysis of covariance (ANCOVA) was used to assess the impact of the EPaL intervention programme on the measures between groups (IG and CG) at Post I and Post II.

**Results:**

The IG reported significantly higher knowledge scores at both Post I (adjusted mean difference = 3.34; 95% confidence interval [CI] = 0.99, 5.69; *p* = 0.006) and Post II (adjusted mean difference = 2.82; 95% CI = 0.86, 4.78; *p* = 0.005) compared with the CG. No significant differences between the IG and CG were found at either Post I or Post II in attitudes, practices, zBMI, WC and BF%. The proportion of participants who were overweight or obese was consistent from Pre to Post II in the IG (35.3%) and increased from 26.2% at Pre to 28.5% at Post II in the CG, but the difference was not statistically significant. The proportion of participants who had abdominal obesity in the IG decreased from 17.6% at Pre to 14.7% at Post II and increased from 16.7% at Pre to 21.4% at Post II in the CG, but the differences were not statistically significant.

**Conclusion:**

Despite no significant reduction of body composition, this programme shows the positive effect on the adolescents’ knowledge regarding healthy lifestyle. This study contributes to the evidence on the effectiveness of school-based health interventions in Malaysian adolescents.

**Trial registration:**

UMIN Clinical Trial Registration UMIN000024349. Registered 11 October 2016.

## Background

Adolescence is a period involving changes in all areas of life – physical, psychological and social [1, 2]. This stage of life is recognised as a second chance at catching up on growth before entering adulthood but is also the stage when body image problems develop as a result of changes associated with physical maturation [1] and eating disorder symptoms first emerge [2]. Weight-related problems, including obesity and disordered eating behaviours, such as unhealthy weight control practices and binge eating, have become a major public health issue in adolescents [3].

Obesity and overweight, defined as an abnormal or excessive fat accumulation that may impair health [4], has been identified as a serious health concern worldwide, where the prevalence of obesity has doubled every 5 to 10 years [5]. Globally, more than 340 million children and adolescents aged 5–19 years were overweight or obese in 2016 [5]. In Malaysia, the National Health and Morbidity Survey 2015 found that the prevalence of obesity among adolescents in Malaysia was 14.4% in adolescents aged 10–14 years and 9.6% in adolescents aged 15–19 years [6]. Furthermore, an alarming increase of about 10% in the prevalence of overweight and obesity among Malaysian adolescents within a single decade, from 18.1% in 2006 [7] to 27.8% in 2018 [8], has been demonstrated in the literature. Immediate actions should be taken to prevent such further increases, as childhood and adolescent obesity problems have adverse consequences, including premature mortality and physical morbidity, later in life [9].

Eating disorders, including anorexia nervosa, bulimia nervosa and binge-eating disorder, affect a much smaller percentage (1–3%) of the adolescent population; however, one-third of the adolescents who exhibit disordered eating behaviours do not meet the criteria for an eating disorder [10, 11]. Disordered eating is not a clinical diagnosis but involves a pattern of eating that can lead to eating disorders. The signs and symptoms of disordered eating include 1) frequent dieting, anxiety associated with specific foods or meal skipping; 2) chronic weight fluctuations; 3) rigid rituals and routines surrounding food and exercise; 4) feelings of guilt and shame associated with eating; 5) preoccupation with food, weight and body image that negatively impacts quality of life; 6) a feeling of loss of control around food, including compulsive eating habits; and 7) using exercise, food restriction, fasting or purging to ‘make up for bad foods’ consumed [12]. The term ‘disordered eating’ is also used to describe dieting and unhealthy weight loss behaviours [13]. In Malaysia, the problem of disordered eating should not be underestimated. Soo et al. [14] reported that 22.3% of female adolescents in Kelantan had disordered eating, whereas a study by Farah Wahida et al. [15] reported that 27.8% of adolescents in Kuantan, Pahang, had disordered eating. Other studies have also reported high prevalence of disordered eating of 18.5% in Sarawak [16] and 19.8% in Selangor [17].

In response to this growing problem, the ‘Eat Right, Be Positive About Your Body and Live Actively’ (EPaL) intervention programme was developed to prevent overweight and disordered eating in Malaysian adolescents. This intervention programme emphasises three components of a healthy lifestyle, namely, healthy eating, positive body image and active lifestyle. The present study aimed to evaluate the effectiveness of the EPaL programme on knowledge, attitudes and practices (KAP) supporting a healthy lifestyle, as well as body composition (body mass index z-score [zBMI], waist circumference [WC] and body fat percentage [BF%]), among adolescents. We hypothesised that the EPaL intervention programme would result in significant improvements in knowledge, attitudes and practices supporting a healthy lifestyle and reductions in zBMI, WC and BF%.

## Methods

### Study design and participants

A quasi-experimental design was used to study adolescents from two schools randomly selected from the district of Hulu Langat in Selangor state, which was provided in the website of the Department of Education of Selangor. This study design involves the use of intact group, which was school that matched with the comparison group on demographic and other key variables [18]. Randomisation was unable to be applied at participants’ level since it may disrupt the classroom activities, as well as this intervention study involved high commitment and cooperation of the school and students. This study design was also been used to avoid contamination [19]. In other words, the participants for intervention and comparison groups were from different schools in order to avoid potential interaction between both groups. Any interaction between these groups may affect the outcome of the effectiveness of the intervention programme. In the present study, the distance between the intervention and comparison schools was about 30 km apart.

The inclusion criteria for the selected schools were that they be coeducational, multiracial, nonresidential and nonreligious. For the participants, they should be students in Form 1 and Form 2 (aged 13 and 14 years) and had consent from parents. Adolescents from one school were allocated to the intervention group (IG) and adolescents from the other to the comparison group (CG), which did not receive any intervention. It should be noted that, although the adolescents from the CG did not receive any study intervention, they continued to attend the standard Health and Physical Education class at least once a week. After the completion of the intervention study, the CG received the intervention modules and educational materials. All Form 1 and Form 2 students were recruited by giving them the respondent’s information sheet and consent letter to be read and completed by the adolescents and their parents prior to enrolment in the study. The participants took part in three points of assessment: pre-intervention (Pre), post-intervention (Post I, after the final session of the intervention) and follow-up (Post II, 3 months after intervention).

### Sample size calculation

Sample size was calculated using the formula for experimental study proposed by Lemeshow et al. [20]. In this calculation, the means of BMI were taken for both intervention and comparison groups from a study by Melnyk et al. [21]. After considering for non-response and non-compliance rate in this study, an additional 30% [22, 23] was added to the sample size. This resulted in 22 participants were needed for intervention and comparison groups, respectively. Hence, the total participants needed for this study was 44 participants.

### Ethical approval and permission

This intervention study was approved by the Ethics Committee for Research Involving Human Subjects of Universiti Putra Malaysia, Selangor, Malaysia (Ref: UPM/TNCPI/RMC/1.4.18.1(JKEUPM)/F2). Permission for data collection was obtained from the Ministry of Education of Malaysia and the State Department of Education of Selangor. Prior to data collection, written consent was obtained from the board and principals of the schools, the adolescents and their parents.

### The EPaL intervention

The study protocol of the EPaL intervention programme was published previously [24]. The EPaL intervention modules [25, 26] and activity book [26] have also been published. The EPaL is a peer-led, school-based health promotion programme developed for secondary school adolescents. EPaL empowers adolescents to live healthier by providing them with knowledge, as well as encouraging positive attitudes towards three components of a healthy lifestyle: healthy eating, positive body image and active lifestyle. The EPaL programme was designed to provide students with cognitive and behavioural skills, which aimed to promote active lifestyle, positive body image and enhance eating behaviours (such as eating at all meal times, decrease fast food consumption and consumption of food-away-from-home, and increase family meal consumption). This in turn will contribute to the decrease in BMI z-score, body fat percentage and waist circumference, reduces disordered eating behaviour and improves health-related quality of life. The EPaL programme consists of eight topics that were delivered by the peer educators over eight sessions within 16 weeks, with 2-week gaps between each session. The eight topics were provided in the study protocol previously [24]. Each session took about 60 to 90 min, depending on the respective topic, delivered during the students’ co-curriculum period at the school hall in EPaL Club. All sessions were conducted with the guide provided in the EPaL Educational Module and monitored by the researchers.

### Measures

#### Socio-demographic characteristics

The socio-demographic characteristics obtained from the participants included sex, ethnicity, age and date of birth.

#### Knowledge, attitude and practice on healthy lifestyle

Knowledge, attitude and practice on healthy lifestyle, with a focus on healthy eating, physical activity and body image among adolescents, were determined using the Knowledge, Attitudes and Practices of EPaL Lifestyle Questionnaire (KAP-ELQ), which was developed by the research team prior to data collection. The instrument comprised items that were adapted from previously published questionnaires, literature reviews and textbooks. The initial items were reviewed for suitability, relevance and accuracy by expert panels comprising nutritionists and health educators. Based on the feedback and recommendations by the expert panels, the items were either retained unchanged, revised or removed.

The final version of the KAP-ELQ that was used in this study consisted of 32 multiple choice questions on knowledge items. For attitudes, there were 24 items with six response categories ranging from 1 (strongly disagree) to 6 (strongly agree). For practices, the participants responded to their practices for the past 7 days. There were 35 practice items, in which 13 had five response categories, ranging from 1 (every day) to 5 (never), and the other 22 practice items had five response categories ranging from 1 (very often) to 5 (never). The ranges of the possible total scores were as follows: knowledge (0–32), attitude (24–144) and practice (35–175). Higher scores in the respective scales indicate better knowledge of a healthy lifestyle, more positive attitudes towards a healthy lifestyle and a healthier lifestyle being practised.

Internal consistency reliability of the KAP-ELQ was determined in this study. For knowledge, the Kuder–Richardson 20 (KR-20) values were 0.54 (Pre), 0.78 (Post I) and 0.69 (Post II). For attitude, Cronbach’s alpha values for the current study were 0.74 (Pre), 0.70 (Post I) and 0.80 (Post II). For practice, Cronbach’s alpha values for the current study were 0.71 (Pre), 0.73 (Post I) and 0.81 (Post II).

#### Anthropometric measurements

Anthropometric measurements were carried out by trained personnel. Body weight was measured using a Tanita Glass Digital Bathroom Scale Model HD-382 (Tanita Corporation, Japan), whereas height was measured with a SECA 206 height mechanical measuring tape (SECA, Germany) to the nearest 0.1 kg and 0.1 cm. Body weight and height were used for BMI calculations according to the formula BMI = weight (kg) / height^2^ (m^2^). The BMI-for-age z-score (zBMI) was determined from the BMI and height values of each participant. The zBMI values were used to categorise body weight status in line with WHO Growth Reference for 5–19 years of age [27]. The zBMI cut-off points were as follows: severe thinness: ≤3 standard deviation (SD); thinness: ≤2 SD; normal weight: ≤2SD and ≥ 1SD; overweight: ≥1 SD; and obesity: ≥2 SD. WC was measured by using a measuring tape to the nearest 0.1 cm. WC was measured at the end of several consecutive natural breaths, at the level parallel to the floor, midpoint between the top of the iliac crest and the lower margin of the last palpable rib in midaxillary line [28]. Abdominal obesity status was determined by referring to the cut-off point of the 90th percentile of WC for Malaysian adolescents aged 12–16 years [29]. An Omron HBF-306 Body Fat Analyzer Scale (Omron Corporation, Japan) was used to measure the percentage of body fat (BF%). The instruments used were adequately calibrated, and adolescents were asked to remove their shoes and empty their pockets before measurement. To minimize the potential adolescent embarrassment, all measurements were carried out in small groups and separately by sex.

### Statistical analyses

The evaluation of the intervention was based on a per-protocol analysis (modified intention-to-treat analysis) [30–32], which was used to retain participants in this study. The criteria for including IG participants in the data analysis were:

1. Compliance with ≥80% of the intervention (attended ≥6 topics); and
2. Completed Pre data collection and at least one post-intervention data collection (Post I or Post II).

For the CG, the criteria for including participants in data analysis were completion of Pre data collection and at least one post-intervention data collection (Post I or Post II).

Initially, 154 adolescents were invited into the IG and 171 adolescents into the CG, of whom 130 in the IG and 71 in the CG consented to participate in the study. In the IG (*n* = 130), 54.6% received less than six topics of the intervention, 13.8% did not receive any intervention, and 5.4% did not complete Post I and Post II data. In the CG (*n* = 71), 40.8% did not complete Post I and Post II data. Our final sample size was 76 adolescents (IG, *n* = 34; CG, *n* = 42), all of whom were included in the final data analysis.

Data were analysed using the IBM SPSS Statistics version 21 software (IBM SPSS Statistics, Inc., Chicago, IL, USA). Descriptive statistics for continuous data are presented using mean and SD. Categorical data are presented as frequencies and percentages. Missing data were treated using the Last observation carried forward (LOCF) method. Independent samples t-test was used to determine differences between the groups (IG and CG) at Pre for continuous variables. Chi-square test was used to determine the association between categorical variables at the pre-intervention. Repeated measures analysis of covariance (ANCOVA) was used to assess the impact of the EPaL intervention programme on the measures between groups (IG and CG) at Post I and Post II. Pre-intervention, age and sex were included as covariances for the analysis of all variables. Cochran’s Q test was used to determine the change in the proportion of participants on body weight status and abdominal obesity status across the three time points. The partial eta-squared value was used to determine the effect size to examine the magnitude of the intervention’s effect. Cohen’s guideline was used to interpret the value as follows: 0.01 = small effect, 0.06 = moderate effect and 0.14 = large effect [33]. The *p*-value of 0.05 was taken as the level of significance.

## Results

### Characteristics of the participants

Table 1 shows the characteristics of the participants at Pre. A majority of the participants was female in the IG (67.6%) and male in the CG (64.3%). All participants were Malays with a mean age of 13.41 years (SD = 0.50) in the IG and 13.14 years (SD = 0.35) in the CG. There were significant differences in sex (*p* = 0.011), age (*p* = 0.010), knowledge score (*p* < 0.001) and body fat percentage (*p* = 0.017) between the IG and CG at Pre. The prevalence of overweight and obesity were 11.8 and 23.5%, respectively in the IG and 4.8 and 21.4%, respectively in the CG. The prevalence of abdominal obesity was 17.6% in the IG and 16.7% in the CG.

**Table 1:** Characteristics of the participants at pre-intervention (*n* = 76)

Characteristics	**IG (*n*** **= 34)**	**CG (*n*** **= 42)**	***p*-value**
Sex			
Male	11 (32.4)	27 (64.3)	0.011*
Female	23 (67.6)	15 (35.7)	
Age (years)	13.41 ± 0.50	13.14 ± 0.35	0.010*
Knowledge score (range = 0–32)	15.03 ± 3.22	12.12 ± 3.62	< 0.001*
Attitude score (range = 24–144)	108.97 ± 10.79	104.10 ± 11.37	0.061
Practice score (range = 35–175)	112.00 ± 11.73	108.26 ± 11.72	0.171
Weight (kg)	48.9 ± 14.0	46.6 ± 16.3	0.513
Height (cm)	151.8 ± 7.6	151.5 ± 8.7	0.868
Waist circumference (cm)	69.3 ± 9.7	67.3 ± 14.3	0.496
Body fat percentage (%)	25.2 ± 6.8	21.5 ± 6.3	0.017*
Body mass index (kg/m^2^)	20.94 ± 4.72	19.94 ± 5.62	0.413
BMI z-score	0.48 ± 1.45	0.11 ± 1.86	0.342
Body weight status			
Severe thinness	0 (0)	2 (4.8)	0.558
Thinness	1 (2.9)	2 (4.8)	
Normal weight	21 (61.8)	27 (64.3)	
Overweight	4 (11.8)	2 (4.8)	
Obesity	8 (23.5)	9 (21.4)	
Abdominal obesity status			
Abdominal obesity	6 (17.6)	7 (16.7)	1.000
Non-abdominal obesity	28 (82.4)	35 (83.3)	

Data are presented as n (%) or mean ± standard deviation

*Significant difference (*p* < 0.05)

### Changes in KAP on healthy lifestyle

#### Between-group differences

Mean scores for KAP on healthy lifestyle in the IG and CG, and the between-group differences at Post I and Post II, are presented in Table 2. The IG reported a significantly higher knowledge score compared with the CG at both Post I (adjusted mean difference = 3.34; 95% CI =0.99, 5.69; *p* = 0.006) and Post II (adjusted mean difference = 2.82; 95% CI = 0.86, 4.78; *p* = 0.005). By contrast, no significant differences in attitude and practice scores were found between the IG and CG at Post I or Post II.

**Table 2:** Mean scores and between-group differences for KAP supporting a healthy lifestyle (*n* = 76)

**Variable**^a^	**Pre**	**Post I**					**Post II**				
	**Mean (SD)**	**Adjusted mean** **(95% CI)**^**b**^	**Adjusted mean difference** **(95% CI)**^**c**^	**F**	***p*-value**	**Adjusted effect size**^**d**^	**Adjusted mean** **(95% CI)**^**b**^	**Adjusted mean difference** **(95% CI)**^**c**^	**F**	***p*-value**	**Adjusted effect size**^**d**^
**Knowledge**											
IG	15.03 (3.22)	18.05 (16.36, 19.75)	3.34 (0.99, 5.69)*	8.03	0.006	0.10 (M)	18.02 (16.61, 19.44)	2.82 (0.86, 4.78)*	8.26	0.005	0.11 (M)
CG	12.12 (3.62)	14.72 (13.27, 16.16)					15.20 (14.00, 16.40)				
**Attitude**											
IG	108.97 (10.79)	107.43 (104.08, 110.77)	−0.30 (−4.86, 4.25)	0.02	0.895	0.00 (N)	107.78 (103.80, 111.76)	2.66 (−2.76, 8.07)	0.96	0.331	0.01 (S)
CG	104.10 (11.37)	107.73 (104.82, 110.64)					105.12 (101.66, 108.58)				
**Practice**											
IG	112.00 (11.73)	106.92 (103.40, 110.44)	−3.95 (−8.73, 0.83)	2.72	0.103	0.04 (S)	108.13 (103.67, 112.59)	−3.23 (−9.28, 2.82)	1.14	0.290	0.02 (S)
CG	108.26 (11.72)	110.87 (107.82, 113.92)					111.37 (107.50, 115.23)				

*Significant difference (*p* < 0.05)

^a^Score ranges: knowledge (0–32), attitude (24–144), practice (35–175). Higher scores in the respective scales indicate better knowledge of a healthy lifestyle, more positive attitudes towards a healthy lifestyle and a healthier lifestyle being practised

^b^Adjusted mean using analysis of covariance after controlling for age, sex and pre-intervention variables

^c^Bonferroni adjustment for 95% confidence interval for differences

^d^Effect sizes: 0.01 = small; 0.06 = moderate; 0.14 = large (Cohen, [34]); N = negligible, S = small; M = moderate

#### Within-group differences

Changes in KAP on healthy lifestyle within each group throughout the study are shown in Table 3. The IG reported significantly higher knowledge scores at Post I (adjusted mean difference = 3.71; 95% CI = 1.73, 5.68; *p* < 0.001) and Post II (adjusted mean difference = 3.82; 95% CI = 2.07, 5.58, *p* < 0.001) compared with those at Pre. However, there were no significant changes in knowledge score between Post I and Post II and no significant changes at all in the attitude and practice scores in the IG. In the CG, significantly higher knowledge scores were reported at Post I (adjusted mean difference = 1.81; 95% CI = 0.10, 3.52; *p* = 0.035) and Post II (adjusted mean difference = 2.24; 95% CI = 0.51, 3.97; *p* = 0.007) compared with those at Pre. There were no significant changes in knowledge score between Post I and Post II, and, similar to IG, there were no significant changes in the attitude and practice scores in the CG.

**Table 3:** Changes in KAP supporting a healthy lifestyle throughout the study (*n* = 76)

**Variable**^**a**^		**IG (*n*** **= 34)**		**CG (*n*** **= 42)**	
	**Difference**	**Mean difference (95% CI)**^**b**^	***p*-value**	**Mean difference (95% CI)**^**b**^	***p*-value**
**Knowledge**	Post I – Pre	3.71 (1.73, 5.68)*	< 0.001	1.81 (0.10, 3.52)*	0.035
	Post II - Pre	3.82 (2.07, 5.58)*	< 0.001	2.24 (0.51, 3.97)*	0.007
	Post II – Post I	0.12 (−1.76, 1.99)	1.000	0.43 (−0.85, 1.71)	1.000
**Attitude**	Post I – Pre	−0.53 (−5.20, 4.14)	1.000	2.83 (−1.73, 7.39)	0.385
	Post II - Pre	−0.15 (−5.39, 5.09)	1.000	0.05 (−5.36, 5.45)	1.000
	Post II – Post I	0.38 (−4.17, 4.94)	1.000	−2.79 (−6.80, 1.23)	0.272
**Practice**	Post I – Pre	2.85 (−7.64, 1.94)	0.428	2.48 (−1.82, 6.77)	0.472
	Post II - Pre	−1.38 (−6.64, 3.88)	1.000	3.14 (−2.66, 8.94)	0.550
	Post II – Post I	1.47 (−1.40, 4.34)	0.616	0.67 (−4.05, 5.38)	1.000

*Significant difference (*p* < 0.05)

^a^Score range: knowledge (0–32); attitude (24–144); practice (35–175). Higher scores in the respective scales indicate better knowledge of a healthy lifestyle, more positive attitudes towards a healthy lifestyle and a healthier lifestyle being practised

^b^Pairwise comparisons with Bonferroni adjustment for 95% confidence interval for the difference

### Changes in body composition

#### Between-group differences

Table 4 shows the mean values for BMI z-score, WC and BF% in the IG and CG and between-group differences at Post I and Post II. No significant differences in zBMI, WC and BF% were found between the IG and CG at Post I and Post II.

**Table 4:** Mean values and between-group differences for body composition (*n* = 76)

**Variable**	**Pre**	**Post I**					**Post II**				
	**Mean (SD)**	**Adjusted mean** **(95% CI)**^**a**^	**Adjusted mean difference** **(95% CI)**^**b**^	**F**	***p*-value**	**Adjusted effect size**^**c**^	**Adjusted mean** **(95% CI)**^**a**^	**Adjusted mean difference** **(95% CI)**^**b**^	**F**	***p*-value**	**Adjusted effect size**^**c**^
**zBMI**											
IG	0.48 (1.45)	0.21 (0.06, 0.37)	− 0.20 (− 0.41, 0.02)	3.44	0.068	0.05 (S)	0.23 (− 0.05, 0.51)	−0.14 (− 0.53, 0.24)	0.57	0.453	0.01 (S)
CG	0.11 (1.86)	0.41 (0.27, 0.54)					0.38 (0.13, 0.62)				
**WC (cm)**											
IG	69.3 (9.7)	70.9 (69.6, 72.2)	−0.0 (−1.8, 1.7)	0.00	0.973	0.00 (N)	71.6 (69.0, 74.2)	−1.3 (−4.9, 2.3)	0.53	0.467	0.01 (S)
CG	67.3 (14.3)	70.9 (69.8, 72.0)					72.9 (70.6, 75.2)				
**BF%**											
IG	25.2 (6.8)	23.3 (22.4, 24.2)	−1.2 (−2.4, 0.1)	3.39	0.070	0.05 (S)	23.3 (22.4, 24.3)	−0.7.(− 2.0, 0.7)	0.96	0.330	0.01 (S)
CG	21.5 (6.3)	24.4 (23.6, 25.2)					24.0 (23.2, 24.8)				

^a^Adjusted mean using analysis of covariance after controlling for age, sex and pre-intervention variables

^b^Bonferroni adjustment for 95% confidence interval (CI) for difference

^c^Effect sizes: 0.01 = small; 0.06 = moderate; 0.14 = large (Cohen, [34]); N = negligible, S = small

#### Within-group differences

Changes in BMI z-score, WC and BF% throughout the study within the two groups are shown in Table 5. From Pre to Post II, there were decreases in the zBMI in the IG and increases in the zBMI in the CG; however, the changes did not reach statistical significance.

**Table 5:** Changes in body composition throughout the study (*n* = 76)

**Variable**		**IG (*n*** **= 34)**		**CG (*n*** **= 42)**	
	**Difference**	**Mean difference** **(95% CI)**^**a**^	***p*-value**	**Mean difference** **(95% CI)**^**a**^	***p*-value**
**zBMI**	Post I – Pre	− 0.08 (− 0.20, 0.04)	0.295	0.11 (− 0.08, 0.31)	0.462
	Post II - Pre	−0.12 (− 0.28, 0.04)	0.224	0.24 (− 0.20, 0.67)	0.551
	Post II – Post I	−0.04 (− 0.22, 0.15)	1.000	0.12 (− 0.30, 0.55)	1.000
**WC (cm)**	Post I – Pre	2.6 (1.0, 4.2)*	0.001	2.4 (1.0, 3.9)*	< 0.001
	Post II - Pre	2.5 (−0.5, 5.6)	0.132	5.5 (2.4, 8.5)*	< 0.001
	Post II – Post I	−0.1 (−3.1, 2.9)	1.000	3.1 (−0.1, 6.3)	0.065
**BF%**	Post I – Pre	0.1 (−1.0, 1.2)	1.000	1.2 (0.0, 2.3)	0.051
	Post II - Pre	0.5 (−0.6, 1.6)	0.765	0.5 (−1.2, 2.1)	1.000
	Post II – Post I	0.4 (−0.6, 1.3)	1.000	−0.7 (−1.7, 0.3)	0.286

^a^Pairwise comparisons with Bonferroni adjustment for 95% confidence interval (CI) for difference

*Significant difference (*p* < 0.05)

With regard to WC, there was a significantly higher figure in the IG at Post I compared with Pre (adjusted mean difference = 2.6 cm; 95% CI = 1.0, 4.2; *p* = 0.001). However, no significant changes in WC were found between Pre and Post II, nor between Post I and Post II, in the IG.

In the CG, higher WC figures were reported for Post I (adjusted mean difference = 2.4 cm; 95% CI = 1.0, 3.9; *p* < 0.001) as well as Post II (adjusted mean difference = 5.5 cm; 95% CI = 2.4, 8.5; *p* < 0.001) compared with Pre. However, no significant change in WC was found between Post I and Post II.

While there was an increase in the WC in both groups between Pre and Post II, the increment in the CG was higher than that in the IG, and the increment in the CG was statistically significant. For both groups, no significant changes in zBMI and BF% were found throughout the study.

### Changes in the proportions of types of body weight status

**Fig. 1:**
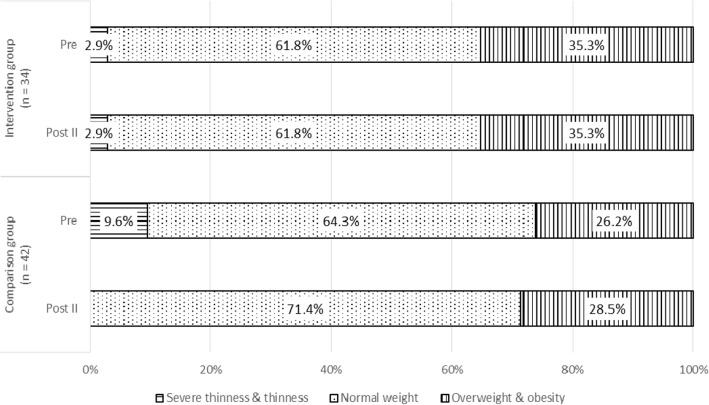
Change in the proportions of types of body weight status among both groups over time

Figure 1 shows the changes in the proportions of types of body weight status among the participants over time. At Pre, a majority of the participants were of normal weight, in both the IG (61.8%) and CG (64.3%). The proportion of the participants who were overweight or obese was slightly higher in the IG (35.3%) compared with that in the CG (26.2%) at Pre. Whereas this proportion held at 35.3% from Pre to Post II in the IG, the proportion of participants who were overweight or obese in the CG group increased from 26.2% at Pre to 28.5% at Post II. However, the increase did not reach statistical significance.

To analyse the changes in the proportions of types of body weight status throughout the study, we re-coded the five categories of body weight status into a two-category (‘non-overweight and non-obesity’ and ‘overweight and obesity’) dichotomy, in which the non-overweight and non-obesity category comprised the ‘severe thinness’, ‘thinness’ and ‘normal weight’ categories, and the overweight and obesity category comprised the ‘overweight’ and ‘obesity’ categories. Cochran’s Q test showed no statistically significant differences for body weight status across the three time points, in both the IG and CG (IG: Q [2, *n* = 34] = 0.000, *p* = 1.000; CG: Q [2, *n* = 42] = 4.667, *p* = 0.097).

### Changes in the proportion of participants with abdominal obesity

**Fig. 2:**
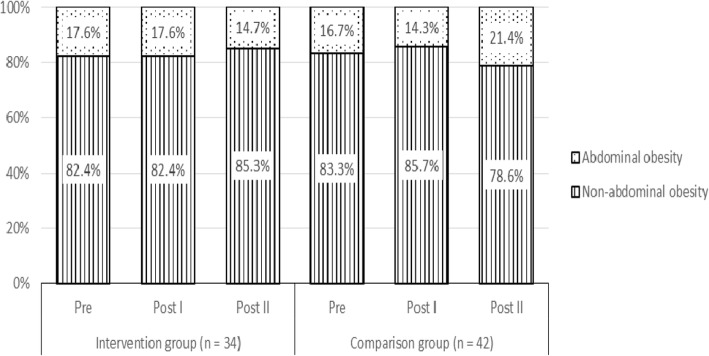
Change in proportion of participants with abdominal obesity status over time

Figure 2 shows the changes in the proportion of participants with abdominal obesity over time. At Pre, most of the participants in both the IG (82.4%) and CG (83.3%) did not have abdominal obesity. In the IG, the proportion of participants with abdominal obesity was maintained at 17.6% at Pre to Post I and then decreased to 14.7% at Post II. In the CG, the proportion of participants who had abdominal obesity decreased from 16.7% at Pre to 14.3% at Post I and then increased to 21.4% at Post II. Cochran’s Q test showed no statistically significant differences for abdominal obesity status across the three time points, in either group (IG: Q [2, *n* = 34] = 0.667, *p* = 0.717; CG: Q [2, *n* = 42] = 3.500, *p* = 0.174).

## Discussion

In this study, adolescents who participated in the EPaL programme showed a markedly greater increase in knowledge scores at Post I and Post II than their control group counterparts, consistent with the results of previous similar studies [34–36]. In the CG, the slight increase in reported knowledge may have been the result of exposure to information regarding healthy lifestyle from other sources, such as their weekly standard Health and Physical Education class, which is compulsory for all students in secondary schools. They could have gained information from the mass media, social media and individuals, such as teachers and parents. Mass media campaigns and social media can produce positive changes, or prevent negative changes, in health-related behaviours across large populations [37] and specifically have the potential to engage adolescents and young adults in their own health [38].

Although there were significant improvements in knowledge in the IG in this study, improvements in attitudes and practices were not statistically significantly different between the two groups of adolescents. Previous studies also showed similar outcomes, i.e. there was a significant change in knowledge about nutrition, but there were no significant changes in nutrition practices or behaviours [34, 39, 40]. Even though nutritional knowledge on its own is not sufficient for behavioural change, an improvement in nutritional knowledge can be considered an important achievement, as it may play a small but crucial role in the adoption of healthier food habits [41].

Based on the Rational model, which is also referred to as the KAP model [42], increasing a person’s knowledge can prompt a behavioural change. The model is based on the premise that the sole obstacle to acting ‘responsibly’ and rationally is ignorance and that information alone will influence behaviour by ‘correcting’ this lack of knowledge. A change in knowledge can cause a change in attitudes/beliefs and thereby cause a change in behaviour; however, this model has weaknesses. While knowledge may be necessary, it is typically not an adequate factor for changing individual or collective behaviour [43]. Motivation is typically derived from sources aside from, or in addition to, factual knowledge. Even though a guide on practising a healthy lifestyle was included in the present study, the programme failed to change the attitudes and practices of the adolescents. This may have been due to the lack of booster sessions, which could have supported additional motivation and increased personal efficacy, leading the adolescents towards healthier lifestyle practices.

The short duration of the intervention also could have been a factor contributing to the few changes observed in participants’ habits or practices. For instance, in promoting healthy eating practices, an increase in understanding and knowledge regarding nutritional concepts does not necessarily bring positive changes in food choices, since people generally need more time to change their food choices and eating habits [44]. Changes in attitude are generally thought to be harder to impact changes in knowledge; however, attitudes are more vital because they are more directly attached to future behaviours. A fairly recent review described an effective classroom-based body image programme that had an average of 5 h in length [45]. Even though our intervention study involved about 8.5 h, there were no changes seen in participants’ attitudes and practices. This could be related to the delivery of intervention by the peer educators, who may have needed more time to influence their peers, as well as to practise being role models for healthy living. More studies are needed to explore the effects of intervention dose and duration on the effectiveness of peer education in improving health-related outcomes among Malaysian adolescents.

Some studies have shown improvements for all aspects of KAP supporting a healthy lifestyle after the implementation of the intervention [46–48]. Involvement of teachers in delivering the intervention programme [47, 48], individual counselling with the intervention subjects [46] and creating a school environment that was aimed at increasing the awareness of healthy nutrition and lifestyle [48] were some keys to the success of previous interventions; none of these were addressed as part of our study.

In our study, zBMI was slightly decreased in the IG and slightly increased in the CG after the programme; however, the changes did not reach statistical significance. The outcomes showed that the intervention contributed to the decrease, albeit nonsignificant, in zBMI among the adolescents. Similarly, nonsignificant decreases in zBMI following the intervention was shown in several other recent studies [34, 49, 50]. The lack of significance in the between-group difference for zBMI in our study contradicts a previous study, in which a significant between-group difference was identified for zBMI at the 10-week follow-up (− 0.24), after 20 weeks of the intervention programme [51]. The lack of a significant change in zBMI in our participants may be partly explained by the inclusion of adolescents across a wide BMI range. In a systematic, meta-analysis review by Peirson et al. [52], preventive interventions in mixed-weight populations of children and adolescents showed small effects in terms of lowered BMI and zBMI, as well as a reduced prevalence of overweight and obesity, in comparison with control groups. Although the mean zBMI of adolescents in the present study was within the normal weight category and is of limited health consequence, we focused on all adolescents regardless of their weight status to emphasise the normative aspects of consuming a healthy diet, practising active lifestyle and having a positive body image.

The proportion of our participants who were overweight or obese was constant (35.3%) in the IG at Pre and Post II. However, in the CG, the proportion increased from Pre to Post II (26.2 to 28.5%). This outcome shows that the prevalence of overweight and obesity was maintained by the exposure of the intervention, even though the prevalence was higher in the IG compared with the CG at Pre. These findings demonstrate the need for this intervention among adolescents to protect them from overweight and obesity. Continuous actions need to be planned and executed to curb this growing problem.

The non-significant changes in the outcome may have been due not only to the short duration of the intervention but also to the short follow-up period, which was not sufficient for meaningful changes in body composition to take place and thus be detectable. Given that our intervention covered three components of healthy lifestyle, longer or more intensive interventions may be needed to achieve substantial changes in outcome variables, such as eating behaviours, physical activity and BMI. Also, adolescents may need more time to learn and apply what they have learned in the intervention programme. There are inconsistencies in the literature with regard to the duration necessary for an intervention programme to be effective in improving the BMI of children and adolescents. In a meta-analysis by Vasques et al. [53], all 52 intervention programmes reviewed obtained significant effects. However, programmes with a duration of 1 year were the most effective (*r* = 0.095), whereas interventions lasting > 1 year produced a smaller effect (*r* = 0.086) [53]. Similarly, a meta-analysis conducted by Cook-Cottone et al. [54] discovered that programmes lasting 28–32 weeks (*r* = 0.07) were more effective than programmes lasting > 32 weeks (*r* = 0.05). A systematic review by Kothandan [55], who reviewed eight family-based and five school-based obesity interventions, concluded that school- and family-based obesity interventions lasting > 6 months showed more significant changes in BMI compared with those lasting < 6 months. These findings might explain why our 16-week intervention did not lead to significant changes in the zBMI and BF%.

Changes in obesity indices, such as the BF% and BMI, are hard to demonstrate in school-based programmes in which all students are considered ‘at risk’ [56]. It is also possible that participants in the intervention had decreases in body fat and increases in lean body mass as a result of their participation in physical activity, without a change in BMI. Moreover, in adolescents, it can be difficult to differentiate the effects of physical activity from expected changes associated with growth and maturation on fat-free mass, when both sexes have significant growth in fat-free mass [57]. In addition, it must be noted that the present intervention was a primary prevention type, not targeted for weight reduction.

The increase in mean WC after the intervention programme was significant in both the IG and CG. However, there was no significant difference between the IG and CG in between-group effect. These outcomes contradict a previous study that showed significant between-group differences for WC after a 20-week post-intervention (− 1.63 cm) [51]. In our study, even though there was an increase in the mean value of WC, the mean WC across the three time points was still within the normal range, whereby the proportion of participants in the IG who had abdominal obesity remained constant from Pre to Post I (17.6%) and decreased to 14.7% at Post II. The reduction in central obesity is particularly important because increased visceral adiposity is associated with a clustering of cardiometabolic risk factors in youth, and its reduction is related to decreases in such risks [58]. There was no significant between-group effect for BF% at either Post I or Post II in the present study. It is important to assess BF% because changes in BF% have health implications, such as changes in blood lipids [59].

The strengths of the present study include the novelties of using a peer-led approach, as well as integrating the prevention of both overweight and disordered eating into a healthy lifestyle intervention programme, in Malaysia. The application of peer education may lead to a dual impact of the programme on the health behaviours of both the participants and the peer educators. Moreover, this dual impact may lead to positive changes in schools and peer norms related to healthy lifestyles. Currently, no other studies have applied a peer-led approach for promoting healthy eating, physical activity or positive body image in Malaysia. To our knowledge, only two health intervention studies have applied this approach, and both were aimed at preventing HIV/AIDS [60, 61]. Ours is also the first health intervention programme in Malaysia that aimed to prevent both overweight and disordered eating, specifically among adolescents.

This study had several limitations. First, it was carried out in one district (Hulu Langat) of Selangor and cannot be generalised to all adolescents from other schools in Selangor or any other state in Malaysia. The results also cannot be generalised to adolescents who are not enrolled in formal education. Secondly, for recruitment of the participants, randomisation was only done at the level of school selection. An experimental design using randomised assignment of participants to groups would produce the highest levels of confidence at which to conclude the validity and causality of the programme’s effectiveness [62]. Although our choice of quasi-experimental design did not involve random allocation, such studies offer the advantage of a real-world situation, helping researchers to understand the complicated nature of the world by enabling them to study environments in real time and in natural settings [63, 64]. Last but not least, the effectiveness of the programme delivery by the peer educators might have varied. Peer educators were likely to have different capabilities in terms of presentation skills, level of participant engagement and personality that affected their delivery of the EPaL topics. Thus, the participants may have had different levels of understanding on the delivered topics based on their peer educator.

## Conclusions

The EPaL intervention programme was effective in improving knowledge supporting a healthy lifestyle among adolescents. Adolescents who participated in the EPaL programme were protected against becoming overweight or obese, or developing abdominal obesity. The outcomes from this study may contribute to the knowledge and evidence on the effectiveness of school-based health interventions and may be used as a model to develop future health and nutrition interventions for adolescents in Malaysia.

## Data Availability

The datasets used and/or analysed during the current study are available from the corresponding author on reasonable request.

## Abbreviations

95% CI: 95% confidence interval
BF%: Body fat percentage
BMI: Body mass index
CG: Comparison group
EPaL: Eat Right, Be Positive About Your Body and Live Actively
IG: Intervention group
KAP: Knowledge, attitudes and practices
KAP-ELQ: Knowledge, Attitudes and Practices of EPaL Lifestyle Questionnaire
SD: standard deviation
WC: Waist circumference
zBMI: Body mass index z-score
